# Abstract of the Proceedings of the Buffalo Medical Association

**Published:** 1865-02

**Authors:** Joseph A. Peters

**Affiliations:** Sec’y.


### ART. IV—*Abstract of the Proceedings of the Buffalo Medical Association.*

TUESDAY EVENING, January 3, 1865.

Society met pursuant to adjournment, the President, Dr. Samo, in the Chair. Present, Drs. White, Rochester, Miner, Gay, Shaw, Congar, Ring, Hauenstein, Johnson and Peters.

The minutes of the last meeting were read and approved.

The Secretary then introduced Dr. H. B. Goff, a practitioner, now attending lectures at the Medical College, who presented an anatomical specimen, with the following explanatory paper, which was ordered to be read:

H. B. P., American by birth, about 50 years of age, the subject of the following report, was an habitual drunkard, and had been gradually sinking, mentally as well as physically, under the influence of alcoholic poisoning for six or seven years. For the last eight months of his life he suffered considerably from chronic diarrhœa and general prostration.

Autopsy was made about twenty-four hours after death. The lungs were found in a normal condition, the mucus coat of the stomach was softened and broken down, the liver healthy, the ventricles of the heart enlarged at the expense of their walls. The immediate cause of his death was evidently inflammation of the bowels, which upon inspection gave the following signs: The bowels were adherent throughout their entire peritoneal extent. The adhesions were of a granular form, varying in size from that of a small shot to that of a common pea, and fastened the convolutions of the bowel, one to another. The kidneys were found in a healthy condition and abnormally connected below the hilus. I have known the patient for twelve years, have been the attending physician of himself and family, and have frequently prescribed for him, but never for any affection of the kidneys or urinary organs, and up to the time of his death the functions of the kidneys were properly performed. The minute anatomy of these kidneys I know nothing about, but will present the specimen for your inspection, hoping at some future time to give you the result of a microscopic examination.

The specimen presented consisted of two kidneys, united at their lower ends by a fibrous band about an inch in width, extending in front of the spinal column, from the internal border of one organ, just below the hilus, to the corresponding portion of the other. Owing to their long maccration in alcohol, little could be judged in regard to their pathological condition, but there was no indication of disease.

*Dr. Peters* had that afternoon attended a coroner's inquest in a case which possessed some points of interest.

Yesterday (January 2d) he had been invited by Dr. Van Guysling to see with him a woman, Mrs. C., who had been taking olium tanaceti, with a probable view to producing abortion. The history of the case as extracted from the patient previous to death, and from witnesses at the inquest was as follows: On Thursday of the preceding week she complained of not feeling very well, but became better, and made a good supper. On Friday and Saturday she was worse, having considerable hemorrhage from the uterus, and a good deal of pain in the abdomen. On Sunday morning she sent for the doctor, who found her with headache, dilated pupils, marked capillary congestion of the face and hands, labored breathing, obstinate vomiting, and a great deal of tenderness over the stomach and intestines. Hemorrhage had nearly or quite ceased. Appropriate remedies were prescribed, and on Monday the abdominal symptoms had ceased, and there had been no return of hemorrhage, though retching and vomiting still continued. The other symptoms were much the same as on Sunday; the pulse feeble, and the tongue dry. The medicines were continued, with slight changes, but this morning the patient died. Post mortem examination showed extensive peritonitis, with the effusion of considerable purulent fluid into the abdominal cavity. The stomach was also congested. but not so much as had been anticipated, and showed one or two points of ulceration. The uterus was firm, and enlarged, measuring about four and a half inches in length by about three in diameter through the fundus. It contained a firm clot, apparently of some days' standing, while about the *os tincæ*, (which have no marks of violence,) were some shreds of more recent clot. The ovaries were congested, and in one the *corpus luteum* of pregnancy was found.

When alive the patient denied having been pregnant, but admitted that she had not menstruated for some two months, and that she had taken the oil to "bring on her courses." No evidence was elicited on the inquest to show that anything resembling a fœtus had escaped before or during the hemorrhage, but neither Dr. Van Guysling nor himself nor the coroner (Dr. Richards) entertained the least doubt that the woman had been pregnant and had aborted. It should be stated that the woman's husband had been dead some months, and she was said to have been living in illicit intercourse with another man.

He (Dr. Peters) had never believed the poison mentioned to have any power over the uterus, and did not now. Thought the abortion to have been the result of the severe shock and vomiting induced. Would call attention to the fact that the drug was said to have been purchased at one of our most respectable druggists, without a prescription. Thought that should be regulated by law.

*Dr. White* considered this to be a highly important subject, and deserving of more attention than it received, but was constrained to believe legislative interference impotent in the matter. The true source of the great prevalence of this sort of crime was to be found in the extremely lax feeling in regard to it in the community; the *sang froid* with which females regarded it was really remarkable. The old idea, still retained in the common law, of the non-vitality of the fœtus previous to quickening was undoubtedly the source of this feeling. Was frank to say that there was no remedy he was acquainted with, which was at all sure to procure abortion. Would be glad to get rid of the idea that ol. tanaceti can produce contraction of the uterus, had never believed it, and should not do so now, though the woman referred to by Dr. Peters undoubtedly aborted after taking the remedy. Thought she must have been at least three or four months pregnant, and had no doubt that the abortion could be accounted for by the great vomiting and disturbance induced by the poisoning. Had again and again known women to take this poison until sick without producing abortion. Even ergot, which increases contraction of the uterus, has, by a wise provision of nature no power to originate it.

*Dr. Rochester* said it was a very common idea that olium tanaceti will procure abortion, but he did not believe it. Knew of one case where a negress took enough to kill her without any abortion. In many cases the effect of these remedies was undoubtedly due to mental impression, probably this was the way the various quack remedies acted. Had attended one woman however who bought Duponco's Golden Pills, took them, and aborted with severe hemorrhage. In applying the tampon, to which he was obliged to have recourse, he discovered the os uteri to be much lacerated, and found the patient had been probing herself with a corset whalebone. This discovery explained why she had so bitterly denounced the pills and the druggist who sold them—they had failed to do their work.

*Dr. Miner* had known several instances where ladies had taken the remedy under discussion, had become sick, had failed to procure any abortion, and had abandoned the drug in disgust. Did not believe that it had any effect different from other essential oils over the uterus. Many women had learned to procure abortion with crotchet needles and similar instruments. Believed physicians sometimes yielded to temptation in such cases; even the professson was not altogether blameless in this matter. He could excuse women who were morally ignorant of the bearings of the case, could excuse even the druggist who sells poisons, etc., indiscriminately, but had no words to express the guilt of the physician who, having been educated, still engages in it. Thought physicians generally did not sufficiently condemn the practice, they were apt to speak jokingly or slightly of it.

*Dr. Congar* had heard respectable medical men named by the people, during the last eight or ten years as procuring abortions, had taken the trouble to watch them and had found it in every instance false.. Physicians were often imposed upon by women. A woman would sometimes call upon a medical man and desire medicine to "make her regular," admitting that she was pregnant, and on his declining to interfere, would consult some other physician, denying the fact of pregnancy, and, perhaps, thus deceiving him into giving some remedy which would result in abortion. In that way physicians might acquire a reputation as abortionists, but did not believe there was a medical man in good standing in this city who would be guilty of it.

*Dr. Rochester* could hardly believe there was any respectable medical man who would be guilty of using mechanical means (to which he understood Dr. Miner to refer) to procure abortion. Had never heard more than two or three medical men mentioned as capable of such things, and they never had any position.

*Dr. Ring*, before coming to Buffalo, had known two men, one an aged man, a student of Dr. Rush of Philadelphia, the other a regularly educated physician, but not a member of any society, who procured abortions openly and fearlessly, but had never known any such case in Buffalo. Was called yesterday to see a lady who had miscarried, and said she brought it on by oil. tanaceti. Such cases undoubtedly sometimes occurred, but were probably attributable to the shock to the system.

*Dr. White* said it must not be forgotten that a large proportion of abortions occur from slight, or other causes connected with the ovum, and not with the mother, that is, were ovuline and not maternal. This would explain abortions often following causes too slight in themselves to produce such a result. Kind hearted practitioners would often, being moved by the distress of pregnant patients, prescribe some placebo, and, abortion taking place, would be accused of procuring it. Did not believe as did Dr. Miner, that any respectable practitioner would be guilty of such practice. Had lived here 32 years, and had known but one case in a circuit of 100 miles; he does it openly, and the consequences are to be seen in our grave-yards.

*Dr. Miner* thought he had been misunderstood. Did not intend to say that a majority, or even any considerable number of respectable practitioners anywhere would do such things. Did believe, however, that physicians had instructed women to procure abortions, and the art of doing so with instruments had been obtained from the profession, and had spread alarmingly. Did not say respectable physicians had done this, but did say graduates of respectable schools, and men in fellowship with the profession. Hoped no one desired to misrepresent him, or make him appear as charging any more upon physicians than facts justified—certainly no one was more ready or anxious than himself to guard the profession from guilt in this matter.

*Dr. Rochester* did not intend to reflect upon Dr. Miner, but certainly thought he had been too sweeping in his statement, if he (Dr. R.) understood him. Got the idea that Dr. Miner meant men of good fellowship, and not one or two sparse individuals.

Some further discussion took place on this point, after which reports were received of prevailing diseases.

*Dr. White* had seen some scarlatina, sore throat, and a little variola. Dr. Rochester had seen some roseola, but no scarlatina. Dr. Gay mentioned diphtheria. Dr. Hauenstein croup, with and without diphtheria. Dr. Johnson varioloid, and diphtheritic croup.

After transacting some miscellaneous business the Association adjourned.

JOSEPH A. PETERS, Sec'y.

